# Adiposity Is Positively Associated With AD-Signature Cortical Thickness in Older Adults

**DOI:** 10.1093/geroni/igaa057.462

**Published:** 2020-12-16

**Authors:** Tina Brinkley, Samuel Lockhart, Bonnie Sachs, Maryjo Cleveland, Benjamin Williams, James Bateman, Samantha Rogers, Suzanne Craft

**Affiliations:** Wake Forest School of Medicine, Winston-Salem, North Carolina, United States

## Abstract

Mid-life obesity is associated with a higher risk for Alzheimer’s disease (AD). However, this association is attenuated or even reversed in late-life, when weight loss may be a preclinical sign of AD. While neuropathological changes likely occur alongside aging-related changes in body composition, this has not been largely investigated. We aimed to determine the association between adiposity and a specific pattern of reduced cortical thickness associated with AD risk and progression. Global and regional adiposity (via dual-energy x-ray absorptiometry) and AD-signature cortical thickness (via surface-based cortical analysis of 3T brain MRI scans) were measured in 35 middle-aged and older adults from the Wake Forest Alzheimer’s Disease Clinical Core (mean age: 69.4±7.8 years, 80% female, 91% White, 29% cognitively impaired). Partial correlations adjusted for age, sex, and cognitive status were examined overall and stratified by age (0.59, p≤0.05). No significant associations were observed in middle-aged adults. These findings suggest that AD-related cortical thinning may be accompanied by a global reduction in body fat among older adults.

